# Metabolomic Fingerprinting of Infants Undergoing Cardiopulmonary Bypass: Changes in Metabolic Pathways and Association With Mortality and Cardiac Intensive Care Unit Length of Stay

**DOI:** 10.1161/JAHA.118.010711

**Published:** 2018-12-07

**Authors:** Jesse A. Davidson, Zachary Pfeifer, Benjamin Frank, Suhong Tong, Tracy T. Urban, Paul A. Wischmeyer, Peter Mourani, Bruce Landeck, Uwe Christians, Jelena Klawitter

**Affiliations:** ^1^ Department of Pediatrics University of Colorado/Children's Hospital Colorado Aurora CO; ^2^ Department of Biostatistics University of Colorado/Children's Hospital Colorado Aurora CO; ^3^ School of Medicine University of Colorado Aurora CO; ^4^ Department of Anesthesiology University of Colorado Aurora CO; ^5^ Department of Research Institute Children's Hospital Colorado Aurora CO; ^6^ Department of Anesthesiology Duke University Durham NC

**Keywords:** congenital heart disease, critical care, kynurenic acid, metabolite, metabolome, methylnicotinamide, neonate, Biomarkers, Metabolism, Clinical Studies, Cardiovascular Surgery, Translational Studies

## Abstract

**Background:**

Mortality for infants undergoing complex cardiac surgery is >10% with a 30% to 40% risk of complications. Early identification and treatment of high‐risk infants remains challenging. Metabolites are small molecules that determine the minute‐to‐minute cellular phenotype, making them ideal biomarkers for postsurgical monitoring and potential targets for intervention.

**Methods and Results:**

We measured 165 serum metabolites by tandem mass spectroscopy in infants ≤120 days old undergoing cardiopulmonary bypass. Samples were collected prebypass, during rewarming, and 24 hours after surgery. Partial least squares–discriminant analysis, pathway analysis, and receiver operator characteristic curve analysis were used to evaluate changes in the metabolome, assess altered metabolic pathways, and discriminate between survivors/nonsurvivors as well as upper/lower 50% intensive care unit length of stay. Eighty‐two infants had preoperative samples for analysis; 57 also had rewarming and 24‐hour samples. Preoperation, the metabolic fingerprint of neonates differed from older infants (*R* ^2^=0.89, Q^2^=0.77; *P*<0.001). Cardiopulmonary bypass resulted in progressive, age‐independent metabolic disturbance (*R* ^2^=0.92, Q^2^=0.83; *P*<0.001). Multiple pathways demonstrated changes, with arginine/proline (*P*=1.2×10^−35^), glutathione (*P*=3.3×10^−39^), and alanine/aspartate/glutamate (*P*=1.4×10^−26^) metabolism most affected. Six subjects died. Nonsurvivors demonstrated altered aspartate (*P*=0.007) and nicotinate/nicotinamide metabolism (*P*=0.005). The combination of 24‐hour aspartate and methylnicotinamide identified nonsurvivors versus survivors (area under the curve, 0.86; *P*<0.01), as well as upper/lower 50% intensive care unit length of stay (area under the curve, 0.89; *P*<0.01).

**Conclusions:**

The preoperative metabolic fingerprint of neonates differed from older infants. Large metabolic shifts occurred after cardiopulmonary bypass, independent of age. Nonsurvivors and subjects requiring longer intensive care unit length of stay showed distinct changes in metabolism. Specific metabolites, including aspartate and methylnicotinamide, may differentiate sicker patients from those experiencing a more benign course.

**Clinical Perspective**

**What Is New?**

- In this study, we demonstrate a profound shift in the metabolic fingerprint of young infants undergoing cardiothoracic surgery with cardiopulmonary bypass that persists through at least 24 hours postoperatively, including a progressive global deficiency in amino acid levels.
- We also identified significant differences in the preoperative metabolic fingerprint of neonates compared with infants 1 to 4 months of age, supporting the concept of metabolic maturation during the first months of life.
- Changes in specific postoperative metabolic biomarkers, including aspartate, glutamate, methylnicotinamide, trigonelline, and kynurenic acid, are independently associated with intensive care unit length of stay and mortality.

**What Are the Clinical Implications?**

- Metabolic fingerprinting and measurement of specific metabolites/metabolic pathways represent novel strategies to elucidate the complex effects of cardiac surgery on infants with congenital heart disease.

## Introduction

Metabolites are low‐molecular‐weight compounds (<1500 Da) that represent the ultimate end products of gene and protein expression.^1^, ^2^, ^3^ The global collection of metabolites, known as the metabolome, maintains dynamic homeostasis and helps determine minute‐to‐minute cellular phenotype.^1^ Recent advances in metabolite analysis allow for simultaneous measurement of hundreds of metabolites from biologic samples. This broad metabolic analysis, or metabolic fingerprinting, can help define unique metabolic patterns and affected pathways present in specific patient groups or disease states.^1^ Metabolic fingerprinting may be particularly informative in the study of acute critical illness because of the dynamic nature of the metabolome compared with upstream tiers of systems biology (genome, transcriptome, and proteome).^1^, ^3^, ^4^

Neonates and young infants undergoing cardiothoracic surgery with cardiopulmonary bypass (CPB) experience substantial physiologic disruption because of a combination of insults, including direct surgical trauma, ischemia‐reperfusion injury, and systemic inflammation.^5^, ^6^, ^7^ This physiologic disruption can, in turn, lead to prolonged periods of critical illness, significant organ injury, and early postoperative mortality.^8^, ^9^, ^10^ The defined period of injury combined with the complex, systemic nature of the disruption makes this pathophysiological condition an ideal candidate for novel metabolomics approaches.

To date, metabolic analysis after pediatric cardiac surgery has been largely limited to single metabolites, most notably lactate,^11^, ^12^ creatinine,^13^ and glucose.^14^, ^15^ Relatively little is known about global metabolic shifts that occur after surgery or their association with postoperative outcomes. A promising pilot study of heterogeneous pediatric cardiac surgery patients found a shift in the untargeted plasma metabolic profile that peaked immediately after surgery, returned toward baseline by 24 hours, and showed moderate predictive capacity for severity of illness and postoperative length of stay (LOS) in the intensive care unit (ICU).^3^ Because of the limited breadth of identified metabolites, this study did not explore potential alterations in metabolic pathways. It also did not assess the neonatal and young infant cardiac surgery population, who generally experience greater postoperative physiologic derangements and are at highest risk for postoperative morbidity and mortality. Expanded metabolic fingerprinting and pathway analysis could provide powerful tools to further define postoperative metabolic derangements in this population and establish early metabolic biomarkers of postoperative morbidity and mortality, particularly in higher‐risk infants.

Our study sought to use targeted metabolic fingerprinting, pathway analysis, and individual biomarker analysis to evaluate metabolic changes in neonates and young infants undergoing cardiothoracic surgery with CPB. Specifically, we aimed to examine differences in preoperative metabolic fingerprints in neonates versus older infants, define the global metabolic shift and metabolic pathway derangements after surgery, and identify individual metabolites capable of serving as early biomarkers of postoperative mortality and ICU LOS.

## Methods

The data, analytic methods, and study materials will be made available to other researchers for purposes of reproducing the results or replicating the procedure. The study data have been made publicly available for review and validation.^16^

### Clinical Cohort

Our study is a secondary analysis of residual frozen serum samples from a recent prospective, observational cohort study of changes in alkaline phosphatase activity in infants undergoing cardiothoracic surgery with CPB. Methods for the parent cohort study have been previously published.^17^, ^18^, ^19^ Briefly, the study enrolled infants ≤120 days of age who were scheduled to undergo clinically indicated cardiothoracic surgery with CPB. Exclusion criteria were weight <2 kg and adjusted gestational age <34 weeks. Residual samples used for the current analysis were drawn preoperatively (after induction of anesthesia and before first surgical incision), during rewarming from CPB but before separation from bypass, and at 24 hours after postoperative admission to the ICU. The primary cohort study also acquired samples at 48 and 72 hours postoperatively, but these draws were of smaller volume and did not result in residual samples. The Colorado Multiple Institution Review Board approved the protocol, and informed written consent was obtained from subjects’ families before enrollment.

### Clinical Data

Baseline perioperative and postoperative clinical data were collected on all subjects, including sex, age at surgery, weight, Aristotle comprehensive complexity score, single‐ventricle physiological feature, preterm delivery, CPB time, cross‐clamp time, and deep hypothermic circulatory arrest/selective cerebral perfusion times. The primary clinical outcome for both the parent cohort study and the current study was occurrence of any of the following major cardiac events: cardiac arrest, need for mechanical circulatory support (extracorporeal membrane oxygenation), death in hospital, or death within 30 days of surgery (inpatient or outpatient). The secondary clinical outcome was ICU LOS.

### Sample Collection and Processing

Serum samples were obtained preoperatively, on rewarming (before separation from CPB), and at 24 hours after return to the ICU. Samples were collected in red‐top tubes and centrifuged at 3000 rpm (1734*g*) for 10 minutes. Then, serum aliquots were placed in standard cryovials and stored at −70°C for batch analysis.

### Metabolomics Assay

Sample analysis was performed on the basis of a previously validated approach using liquid chromatography–tandem mass spectrometry.^20^, ^21^ Samples were analyzed using an Agilent 1200 series high‐performance liquid chromatography (HPLC) system (Agilent Technologies, Palo Alto, CA) interfaced with an ABSciex 5500 hybrid triple quadrupole/linear ion trap mass spectrometer (Concord, ON, Canada) equipped with an electrospray ionization source operating in the positive/negative switch mode.

Briefly, 50 μL serum sample was combined with 400 μL cooled methanol, incubated for protein precipitation overnight, and dried in a SpeedVac concentrator centrifuge (Thermo Fisher Scientific/Savant, Waltham, MA). Reconstitution was performed in 20 μL of water/methanol (80:20) and subjected to a modified targeted metabolomics analysis with relative quantification (165 metabolites in the current assay).

#### Data acquisition

The precursor ion and fragment ion transitions, the metabolite names, dwell times, and the appropriate collision energies for both positive and negative ion modes were adapted from Yuan et al^20^ with several metabolite transitions added by our group. Precursor ion and fragment ion transitions were set to unit resolution for optimal metabolite ion isolation and selectivity. In addition, the polarity switching (settling) time was set to 50 ms; in 1.42 s using a 3‐ms dwell time, we were able to obtain 6 to 14 scans per metabolite peak. The source temperature was set at 500°C, curtain gas (nitrogen) at 20, collision gas (nitrogen) at high, ion source gases 1 and 2 at 33, declustering potential at ±93, entrance potential at ±10, and collision cell exit potential at ±10 for positive and negative ion modes.

Sample (8 µL) was injected onto an Amide XBridge HPLC column (3.5 μm; 4.6‐mm inner diameter×100‐mm length) (Waters, Milford, MA). The mobile phases consisted of HPLC buffer A (pH=9.0: 95% [vol/vol] water, 5% [vol/vol] acetonitrile, 20 mmol/L ammonium hydroxide, 20 mmol/L ammonium acetate) and HPLC buffer B (100% acetonitrile). The HPLC settings were as follows: from 0 to 3 minutes, the mobile phase was kept at 85% B; from 3 to 22 minutes, the percentage of solvent B was decreased from 85% to 2% and was kept at 2% for an additional 3 minutes. At minute 26, solvent B was increased again back to 85% and the column was flushed for an additional 7 minutes at 85% solvent B.

#### Data analysis

Once the data were acquired, MultiQuant, v2.1.1. (ABSciex) software was used for initial data processing. Resulting metabolite peak areas were normalized to the area of the internal standard and tissue weight, and this ratio was used for all subsequent statistical analyses.

### Statistical Analysis

Data distributions were examined before any data analysis. Patients’ demographics and baseline clinical characteristics were summarized using frequency and percentage for binary or categorical variables, whereas median and range were presented for continuous numeric variables. To demonstrate our study population unavailable for follow‐up at random at each sample time point, we compared the study cohort with the cohort without available samples at each stage to take advantage of sample independence. The χ^2^ test was used to compare the proportions of categorical variables, and the Kolmogorov‐Smirnov test was applied to compare the nonnormal distribution of the continuous data. *P*<0.05 was considered as statistically significant. SAS/STAT software V9.4 was used for this clinical data analysis.

Metabolomic statistical analysis was performed using Metaboanalyst 4.0, a comprehensive web‐based metabolomics analysis tool (http://www.metaboanalyst.ca).^22^ Relative peak intensities were initially log transformed and then autoscaled (mean centered and divided by the square root of the SD of each variable). Partial least squares–discriminant analysis (PLS‐DA) was performed as the initial analysis to assess for capacity to discriminate among prespecified groups. This validated multivariable technique allows analysis of multiple analytes to differentiate the metabolic profile of different groups, even when the number of subjects per group is significantly smaller than the number of analytes.^23^, ^24^, ^25^, ^26^ R^2^Y (a measure of goodness of fit) and Q^2^Y (consistency on cross‐validation) are reported for each model.^27^ Empiric *P* values for this analysis were obtained through permutation testing, where class data are randomly reassigned over 2000 permutations and reanalyzed. The *P* value was calculated on the basis of the proportion of times that class separation from random assignment was at least as good as the original supervised class separation.^22^ Variable importance in projection scores were used to identify the top 15 metabolites driving variation among groups. Pathway analysis was performed using the pathway analysis tool in Metaboanalyst. This tool uses both pathway enrichment analysis through the R‐package GlobalTest based on compound concentration values as well as pathway topological analysis accounting for the impact of individual measured metabolites within the pathway. The goal of assessing pathway impact is to account for pathway structure and the intuitive concept that central or nodal positions in a pathway will have a greater impact than marginal or isolated positions. Total or maximal importance for each pathway is designated as 1, whereas the importance of measured metabolites to that pathway is designated as the cumulative percentage from matched metabolite nodes. A total of 56 pathways were analyzed using this tool. Using the Bonferroni correction to adjust for multiple comparisons, pathways with a *P*<0.00089 were considered statistically significant. Biomarker analysis of metabolites was performed using *t* tests as well as univariate and multivariable receiver operator characteristic curve analysis. For multivariate receiver operator characteristic curve analysis, empiric *P* values were generated after permutation testing with random reassignment of group labels (1000 permutations). We also performed univariate and multivariable analysis (JMP Pro V13.1.0) of covariates to assess the independent association of metabolites of interest with clinical outcomes. For the univariate analysis, we chose clinically relevant preoperative, intraoperative, and postoperative variables: age at surgery, weight at surgery, Aristotle comprehensive score,^28^ presence of single‐ventricle physiological feature, CPB time, aortic cross‐clamp time, vasoactive‐inotropic score^29^, ^30^ at 6 hours after operation, peak creatinine, change in creatinine, and Neutrophil gelatinase‐associated lipocalin at rewarming as well as 6 hours after operation. Covariates found to be associated with the outcome of interest on univariate analysis (*P*<0.05) were included in the final multivariable model.

## Results

### Subjects and Clinical Characteristics

A total of 122 patients were enrolled in the parent cohort study, including 2 screen failures, as previously reported.^17^ The sample collection rate was 98.8% through 24 hours postoperation. Eighty‐two subjects had a residual preoperative and rewarming serum aliquot available for metabolomic analysis. The 24‐hour sample was smaller and less often resulted in a residual aliquot available for analysis, leading to a total of 57 subjects with a complete set of residual preoperative, rewarming, and 24‐hour samples available. Baseline and operative characteristics as well as postoperative outcomes are shown in Table 1. There were no significant clinical differences between the parent cohort and the subgroups analyzed for this study. A total of 6 subjects in this study met the primary outcome of death (in hospital or within 30 days), cardiac arrest, or need for mechanical circulatory support; all 6 subjects ultimately died. These subjects demonstrated a range of anatomical and physiological characteristics as well as length of survival (Table 2).

**Table 1:** Baseline, Operative, and Postoperative Characteristics

Clinical Characteristic	Full Cohort (n=120)	Preoperative Sample Available (n=82)	No Preoperative Sample (n=38)	*P* Value	3 Time Points Available (n=57)	No 24‐h Sample (n=63)	*P* Value
Female sex, n (%)	52 (43.3)	37 (45.1)	15 (39.5)	0.56	27 (47.4)	25 (39.7)	0.40
Age at surgery, median (range), d	14.5 (1.0–120)	20 (1–120)	8.5 (2–118)	0.21	11 (1–120)	17 (2–119)	0.95
Preterm, n (%)	16 (13.3)	13 (15.9)	3 (7.9)	0.39	9 (15.8)	7 (11.1)	0.45
Weight, kg, median (range)	3.5 (2.1–7.9)	3.6 (2.1–7.9)	3.5 (2.1–5.4)	0.73	3.5 (2.1–7.9)	3.6 (2.1–7.4)	0.66
Aristotle score,^28^ comprehensive, median (range)	10.0 (3.0–19.5)	10.0 (3.0–19.5)	9.0 (3.0–14.5)	0.63	10.3 (3.0–19.5)	9.0 (3.0–14.5)	0.14
CPB time, median (range), min	137.5 (0–399)	137.5 (54–399)	137 (0–277)	0.29	142 (56–399)	130 (0–277)	0.17
Cross‐clamp time, median (range), min	74.5 (0–241)	75 (0–241)	69.5 (0–205)	0.83	79 (0–241)	69 (0–205)	0.19
Deep hypothermic circulatory arrest, median (range), min	0 (0–154)	0 (0–154)	0 (0–77)	0.54	0 (0–154)	0 (0–77)	0.99
Selective cerebral perfusion, median (range), min	0 (0–115)	0 (0–115)	0 (0–78)	0.99	0 (0–115)	0 (0–82)	1.00
Single‐ventricle physiological feature, n (%)	38 (31.9)	25 (30.9)	13 (34.2)	0.72	20 (35.1)	18 (29.03)	0.48
Mortality, n (%)	9 (7.6)	7 (8.5)	2 (5.4)	0.72	6 (10.5)	3 (4.8)	0.31
Combined primary outcome, n (%)^a^	11 (9.3)	8 (9.9)	3 (8.1)	1.00	6 (10.5)	5 (8.2)	0.66
CICU length of stay, median (range), d	6 (1–42)	6 (1–42)	5.0 (2.8–29)	0.93	6 (1–42)	6 (1–40)	0.49
Vasoactive inotropic score^29^, ^30^ at 24 h, median (range)	7 (0–30)	7.3 (0–30)	7 (0–26)	1.00	8 (0–30)	5.5 (0–26)	0.80
Duration of mechanical ventilation, median (range), h	44.5 (0.1–762.6)	45.2 (0.1–762.6)	44.2 (0.5–453.1)	0.90	43.1 (0.1–762.6)	45.6 (0.5–453.1)	0.80

CICU indicates cardiac intensive care unit; CPB, cardiopulmonary bypass.

a Death in hospital or within 30 days, cardiac arrest, or need for extracorporeal membrane oxygenation.

**Table 2:** Clinical Characteristics of Subjects Meeting the Primary Outcome of Death, Cardiac Arrest, or Need for Mechanical Circulatory Support

Study No.	Diagnosis	Age at Surgery, d	Single‐Ventricle Physiological (Postoperative)	ECMO	Postoperative Day of Death
23	Pulmonary atresia/VSD; TAPVR	3	Yes	No	149
33	Tracheal stenosis; VSD; ASD	60	No	Yes	6
50	DORV; ASD	35	No	No	34
64	DORV; mitral atresia	5	Yes	No	148
98	HLHS; VSD; PAPVR	8	No	No	73
121	D‐TGA; aortic arch hypoplasia	8	No	Yes	42

ASD indicates atrial septal defect; DORV, double‐outlet right ventricle; D‐TGA, d‐transposition of the great arteries; ECMO, extracorporeal membrane oxygenation; HLHS, hypoplastic left heart syndrome; PAPVR, partial anomalous pulmonary venous return; TAPVR, total anomalous pulmonary venous return; VSD, ventricular septal defect.

### Age Differences in Preoperative Metabolic Fingerprint

**Figure 1:**
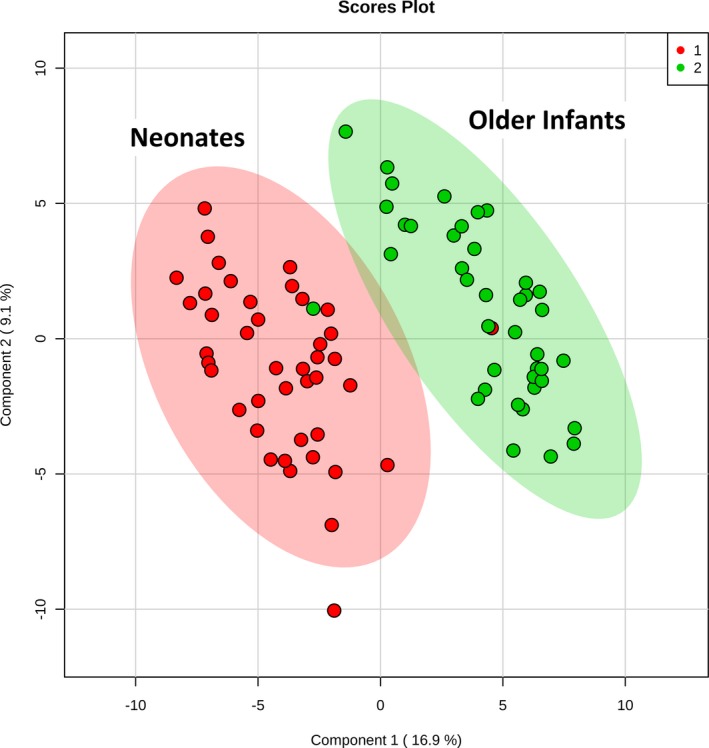
Two‐dimensional partial least squares–discriminant analysis comparing preoperative metabolic fingerprints of neonates (red) vs older infants (green).

**Figure 2:**
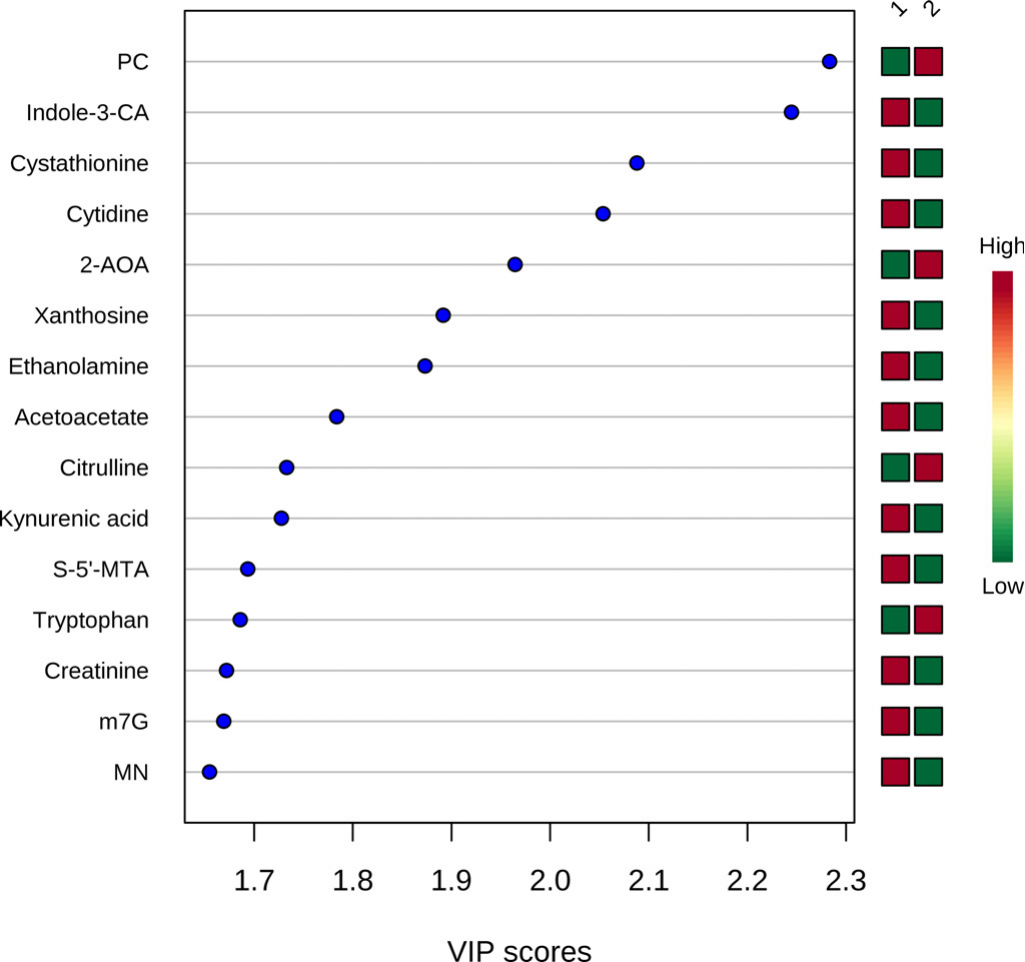
Variable importance in projection (VIP) scores for the top 15 metabolites contributing to variation in metabolic fingerprints of neonates (group 1) vs older infants (group 2). 2‐AOA indicates 2‐aminooctanoic acid; Indole‐3‐CA, indole‐3‐carboxylic acid; m7G, 7‐methylguanosine; S‐5′‐MTA, S‐methyl‐5′‐thioadenosine.

**Figure 3:**
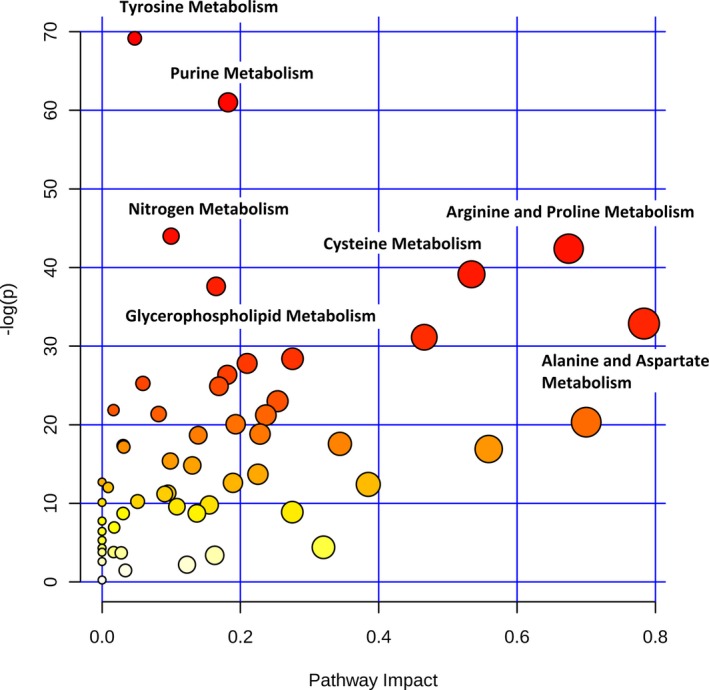
Differences in metabolic pathways between neonates and older infants (preoperative). The *x* axis and size of circles represent importance of differential metabolites within the pathway. The *y* axis and color of circles represent statistical significance of the overall metabolic changes within the pathway.

We first compared the preoperative metabolic fingerprints of neonates and older infants to assess the need to stratify our postoperative analyses by age (n=82). The preoperative metabolic fingerprint of neonates differed markedly from that of older infants on PLS‐DA (R^2^Y=0.89; Q^2^Y=0.79; empiric *P*<0.001) (Figure 1). The top 15 metabolites contributing to variation in metabolic fingerprint between neonates and older infants along with relative concentrations at each time point are shown in Figure 2. Multiple metabolic pathways differed significantly between the 2 ages, most notably tyrosine metabolism (*P*=9.1×10^−31^), purine metabolism (*P*=3.2×10^−27^), nitrogen metabolism (*P*=7.8×10^−20^), and arginine/proline metabolism (*P*=3.9×10^−19^) (Figure 3). From a biomarker perspective, >45 metabolites demonstrated significant differences after correction for multiple comparisons. The top 4 metabolites differentiating neonatal from older infant serum at the preoperative time point were phosphorylcholine (*P*=9.3×10^−18^), indole‐3‐carboxylic acid (*P*=7.0×10^−17^), cystathionine (*P*=7.6×10^−14^), and cytidine (*P*=2.8×10^−13^). These 4 metabolites combined could differentiate between neonates and older infants preoperatively on receiver operator characteristic curve analysis, with an area under the curve (AUC) of 0.99 (empiric *P*<0.01). We also assessed phosphorylcholine against age as a linear variable to see if there was evidence of a continuous change with age. Phosphorylcholine demonstrated a strong positive correlation with age (*r*=0.8) through 120 days of age (the oldest infants in our study).

We next repeated these analyses after dividing the cohort into subjects with all 3 samples available for analysis (derivation cohort: n=57) compared with the group with only preoperative and rewarming samples available (test cohort: n=25) to assess for potential systematic differences between these groups. Both subcohorts demonstrated a substantial difference in the metabolic fingerprint of the neonates compared with the older infants with strong discrimination on PLS‐DA (derivation cohort R^2^Y=0.96, Q^2^Y=0.79; test cohort R^2^Y=0.99, Q^2^Y=0.78). The top metabolites contributing to the variation in both subcohorts were remarkably similar, with 8 of the top 15 metabolites in the derivation cohort also appearing in the top 15 of the test cohort (with identical directionality of change). Of the remaining 7 metabolites from the top 15 of the derivation cohort, 5 shifted only marginally in their importance in the test cohort, ranging from 16th to 26th most important (of 165) in the test cohort. This analysis is consistent with the findings from the previously described permutation testing performed on the full cohort (empiric *P*<0.001) and suggests that the differences in metabolic fingerprints between neonates and older infants at our center are unlikely to be attributable to chance or sampling bias.

### Postoperative Changes in Metabolic Fingerprint

**Figure 4:**
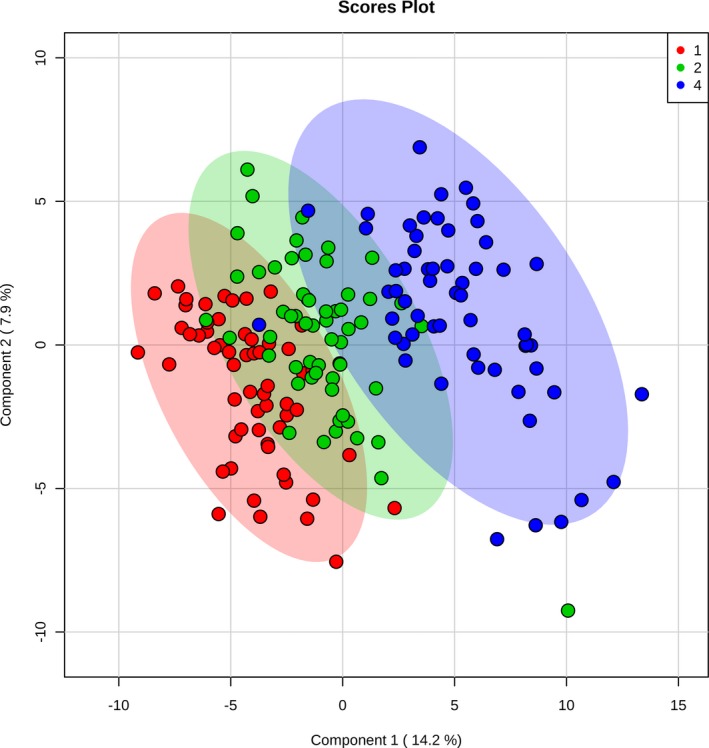
Two‐dimensional partial least squares–discriminant analysis comparing metabolic fingerprints of the complete cohort at baseline (red), rewarming from cardiopulmonary bypass (green), and 24 hours (blue).

**Figure 5:**
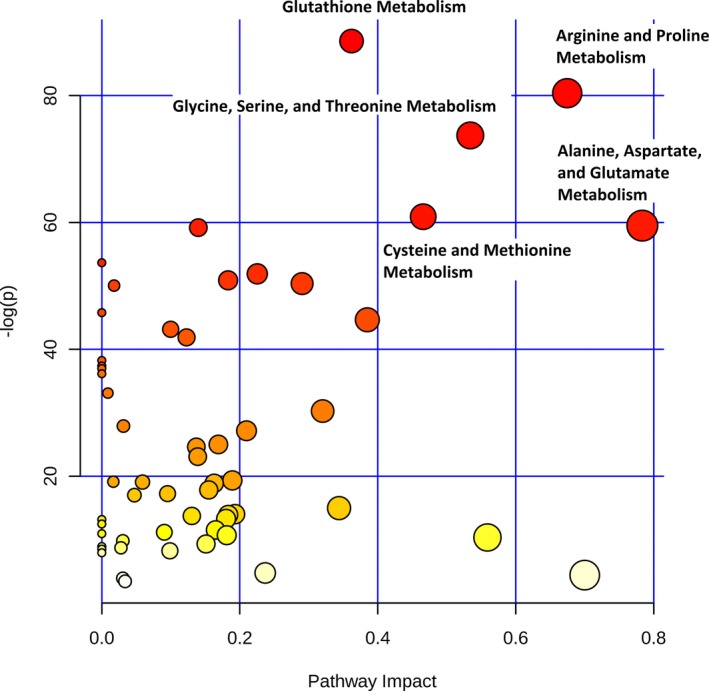
Difference in metabolic pathways among preoperative, rewarming, and 24‐hour time points across the full cohort. The *x* axis and size of circles represent impact of differential metabolites within the pathway. The *y* axis and color of circles represent statistical significance of the overall metabolic changes within the pathway.

**Figure 6:**
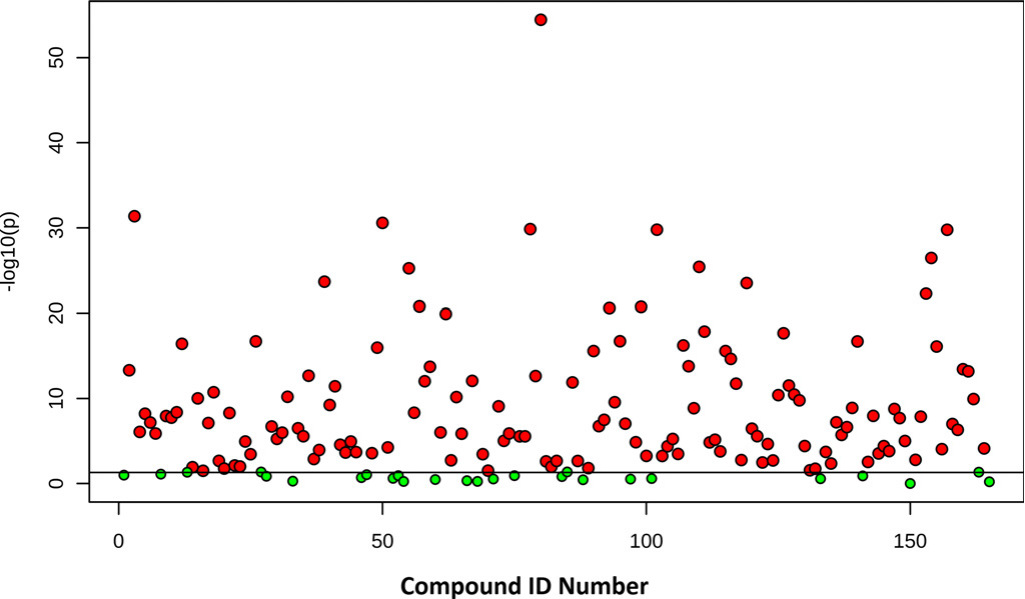
Differences in individual metabolites among preoperative, rewarming, and 24‐hour samples by 1‐way ANOVA. Red=statistically significant at an adjusted *P*=0.05.

**Figure 7:**
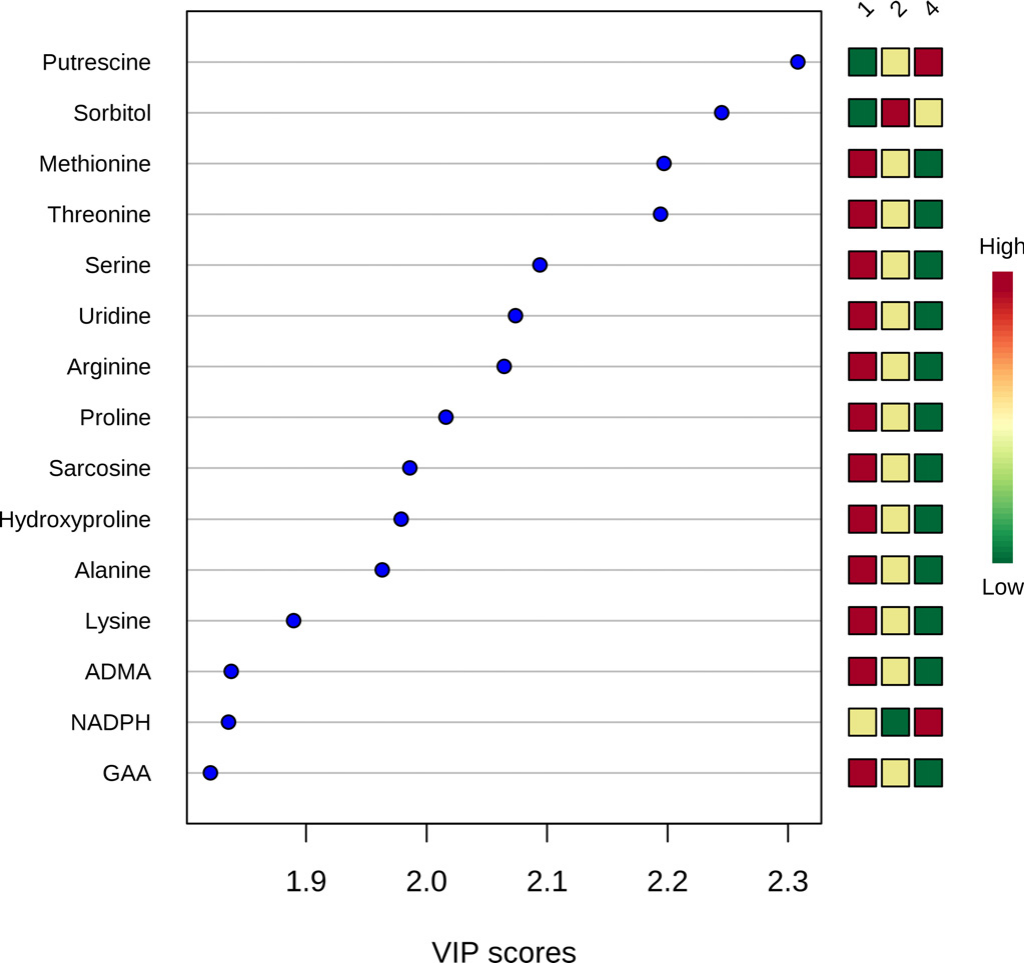
Variable importance in projection (VIP) scores for the top 15 metabolites contributing to variation in postoperative changes in metabolic fingerprint (whole cohort). ADMA indicates asymmetric dimethylarginine; GAA, guanidoacetic acid; NADPH, nicotinamide adenine dinucleotide phosphate.

We next evaluated shifts in the metabolic fingerprint induced by cardiac surgery with CPB. Because of our findings of differences in preoperative metabolic fingerprints between neonates and older infants, we evaluated postoperative changes in both the whole cohort as well as in the neonatal and older infant cohorts alone. In the cohort of subjects with all 3 time points available for analysis (n=57), cardiac surgery with CPB bypass induced a marked shift in the metabolic fingerprint in our children, as demonstrated by PLS‐DA analysis (R^2^Y=0.92, Q^2^Y=0.83; empiric *P*<0.001) (Figure 4). In addition, the metabolic fingerprint continued to diverge from baseline through the 24‐hour time point in almost all subjects. Multiple pathways demonstrated changes, with arginine/proline (*P*=1.2×10^−35^), glutathione (*P*=3.3×10^−39^), glycine/serine/threonine (*P*=9.6×10^−33^), and alanine/aspartate/glutamate (*P*=1.4×10^−26^) metabolism most significantly altered (Figure 5). Of the 165 individual metabolites tested, 139 demonstrated significant changes from preoperation through 24 hours postoperatively after adjustment for multiple comparisons (Figure 6). The top 15 metabolites contributing to variation in metabolic fingerprint after surgery, along with relative concentrations at each time point, are shown in Figure 7. These included postoperative increases in putrescine and sorbitol as well as decreases in both polar and nonpolar amino acids and their derivatives. To confirm that these changes were not likely secondary to sampling bias, we repeated the analysis of the change in metabolic fingerprint from preoperation to rewarming using the subcohort of subjects with only preoperative and rewarming samples available (n=25). PLS‐DA readily distinguished the metabolic fingerprints of preoperative and rewarming samples in this subcohort (R^2^Y=0.96, Q^2^Y=0.73). The top metabolites driving the variation between preoperative and rewarming metabolic fingerprints were identical to those in the subcohort with all 3 samples available (n=57), suggesting a similar metabolic response to CPB in these 2 groups, at least through the rewarming time point.

**Figure 8:**
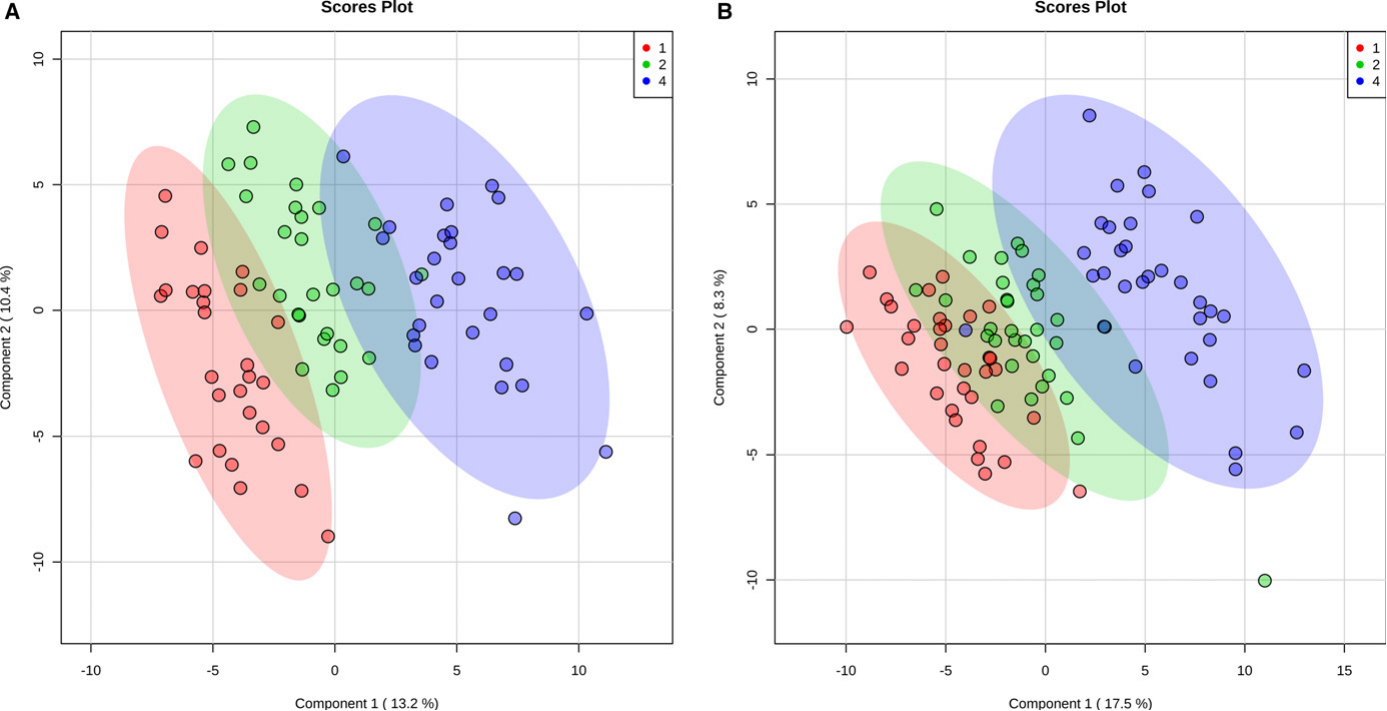
Two‐dimensional partial least squares–discriminant analysis comparing metabolic fingerprints of older infants **(A)** and neonates **(B)** at baseline (red), rewarming from cardiopulmonary bypass (green), and 24 hours (blue).

**Figure 9:**
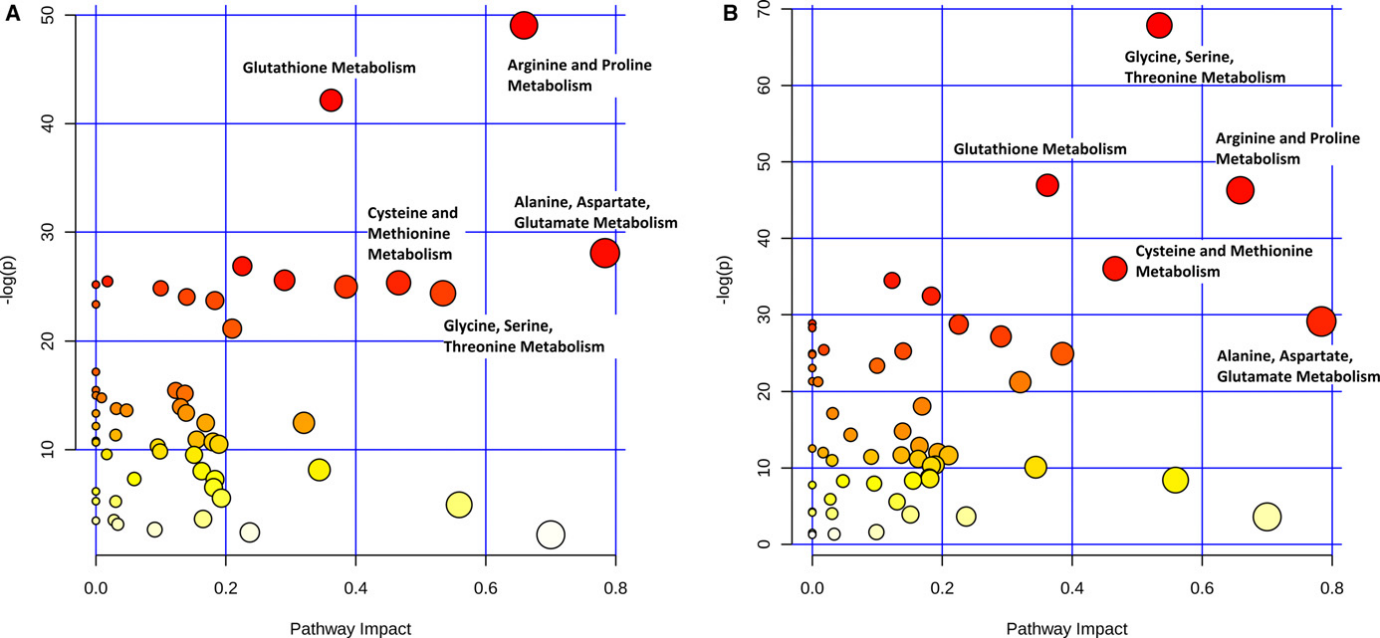
Difference in metabolic pathways among preoperative, rewarming, and 24‐hour time points in older infants **(A)** and neonates **(B)**.

**Figure 10:**
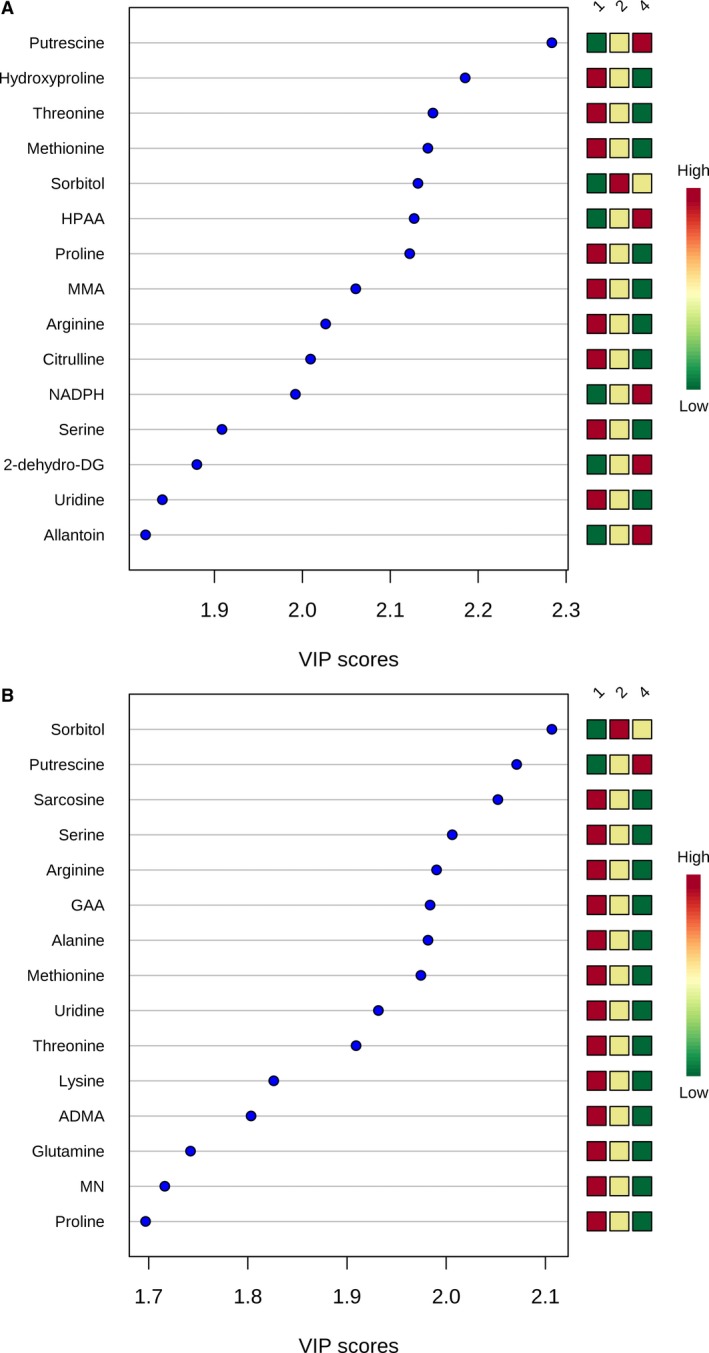
Variable importance in projection (VIP) scores for the top 15 metabolites contributing to variation in postoperative changes in metabolic fingerprint in older infants **(A)** and neonates **(B)**. 2‐dehydro‐DG indicates 2‐dehydro‐d‐gluconate; ADMA, asymmetric dimethylarginine; GAA, guanidoacetic acid; HPAA, hydroxyphenylacetic acid; MMA, methylmalonic acid; NADPH, nicotinamide adenine dinucleotide phosphate.

Neonates and older infants demonstrated similar patterns on PLS‐DA when analyzed separately (Figure 8). Changes in specific pathways were also similar between neonates and older infants (Figure 9), with the most significant alterations found in arginine/proline, glutathione, glycine/serine/threonine, alanine/aspartate/glutamate, and cysteine/methionine metabolism. Of the top 15 metabolites contributing to variation in the metabolic fingerprint of neonates and older infants, 8 were similar in both importance and relative concentration changes in both the neonatal and older infant subcohorts (putrescine, threonine, methionine, sorbitol, proline, arginine, serine, and uridine) (Figure 10).

### Metabolic Fingerprint and Postoperative Outcomes

#### Primary outcome

**Figure 11:**
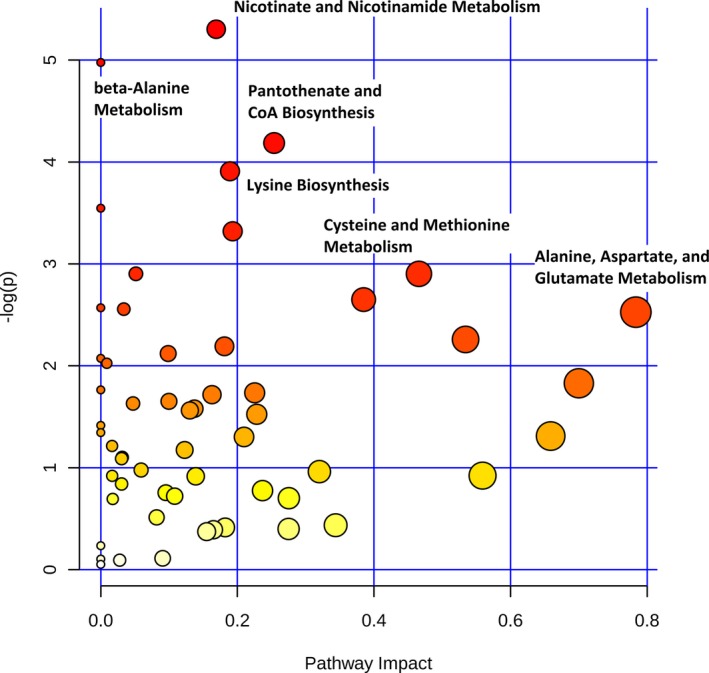
Difference in metabolic pathways between survivors and nonsurvivors.

We next sought to determine if early postoperative (24‐hour) metabolic fingerprinting could be used to discriminate between subjects who did or did not experience the primary outcome of death, cardiac arrest, or need for postoperative extracorporeal membrane oxygenation. Because all 6 subjects who met the primary clinical outcome ultimately died either in hospital or within 30 days of surgery (Table 2), we will subsequently refer to this group as nonsurvivors. PLS‐DA analysis of the 24‐hour metabolic fingerprint was able to discriminate between survivors and nonsurvivors with R^2^Y=0.91 in our cohort but demonstrated overfitting on cross‐validation (Q^2^Y=−0.27), likely because of the small number of subjects in the nonsurvivor group. Metabolic pathways differing between survivors and nonsurvivors are shown in Figure 11. The greatest differences were found in nicotinate/nicotinamide metabolism (*P*=0.005) and aspartate metabolism (including β‐alanine metabolism [*P*=0.008], pantothenate biosynthesis [*P*=0.02], lysine biosynthesis [*P*=0.02], cysteine/methionine metabolism [*P*=0.05], and alanine/aspartate/glutamate metabolism [*P*=0.08]), although these did not meet criteria for statistical significance after correction for multiple comparisons.

**Figure 12:**
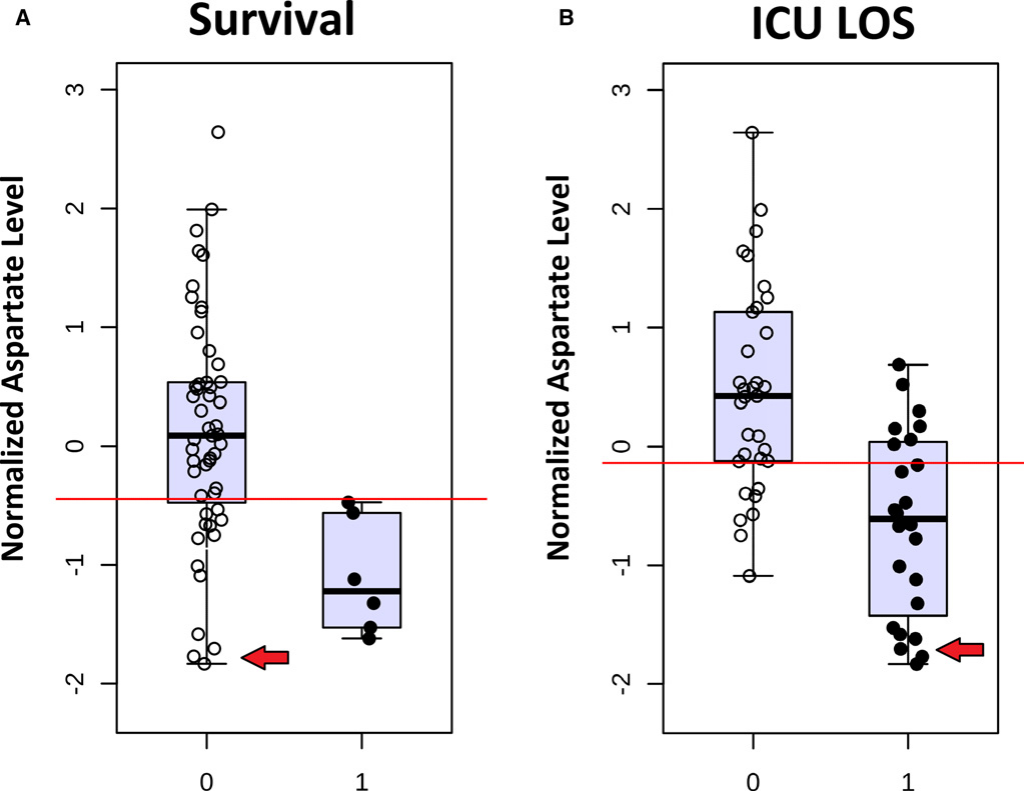
Normalized aspartate levels at 24 hours in survivors (0) vs nonsurvivors (1) **(A)** and lower (0) vs upper (1) 50% of intensive care unit length of stay (ICU LOS) **(B)**. Red arrows indicate subjects with low aspartate levels who survived but with extended ICU LOS.

**Figure 13:**
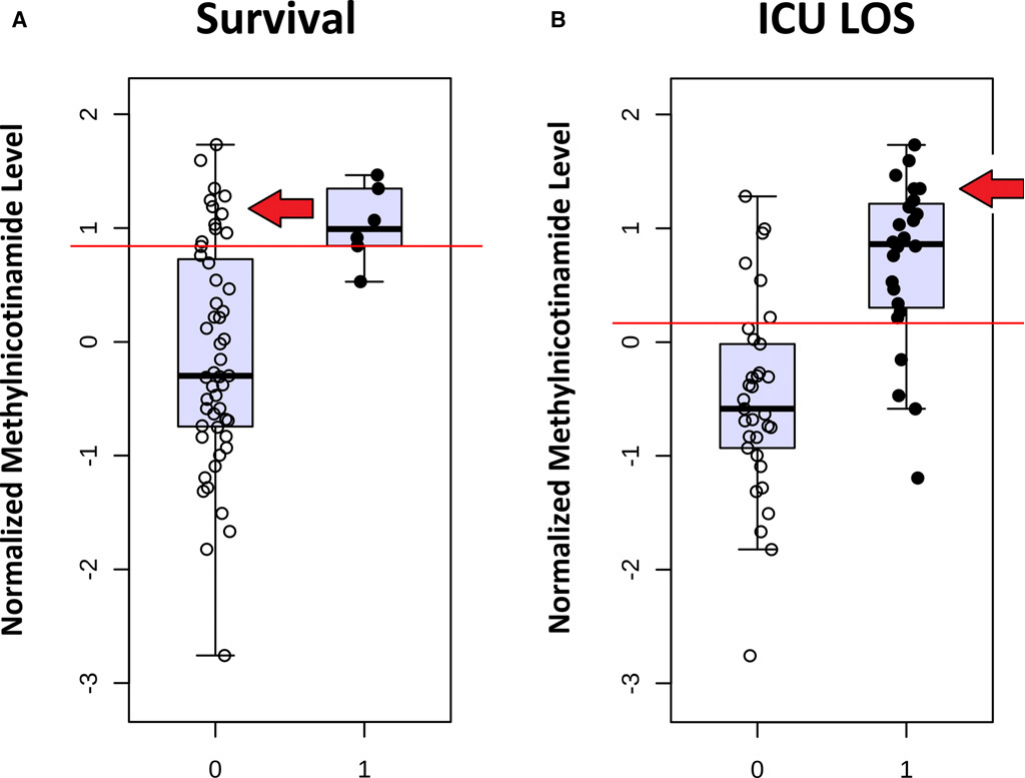
Normalized methylnicotinamide levels at 24 hours in survivors (0) vs nonsurvivors (1) **(A)** and lower (0) vs upper (1) 50% of intensive care unit length of stay (ICU LOS) **(B)**. Red arrows indicate subjects with high methylnicotinamide levels who survived but with extended ICU LOS.

Multiple individual metabolites demonstrated potential ability to discriminate between survivors and nonsurvivors. These metabolites include aspartate (AUC, 0.87) and glutamate (AUC, 0.80) from alanine/aspartate/glutamate metabolism, methylnicotinamide (AUC, 0.84) and trigonelline (AUC, 0.80) from nicotinate/nicotinamide metabolism, as well as kynurenic acid (AUC, 0.80) from tryptophan metabolism. Relative normalized concentrations of the individual metabolites aspartate and methylnicotinamide are shown in Figures 12 and 13. Normalized aspartate levels were significantly lower (*P*=0.003), whereas normalized methylnicotinamide levels were significantly higher (*P*=0.006) in nonsurvivors. Similar differences were found when analyzing neonates alone (aspartate lower [*P*=0.03] and methylnicotinamide higher [*P*=0.05] in nonsurvivors compared with survivors). Using multiple permutation techniques, the combination of aspartate and methylnicotinamide demonstrated the strongest ability to discriminate between survivors and nonsurvivors (AUC, 0.86; empiric *P*<0.01). Again, similar findings occurred when analyzing neonates alone (AUC, 0.82; empiric *P*=0.06). Both aspartate and methylnicotinamide remained independently associated with mortality (*P*<0.05) on multivariable analysis.

#### ICU LOS

Figures 12A and 13A show that although 24‐hour aspartate and methylnicotinamide provided good discrimination between survivors and nonsurvivors in our cohort, some survivors did demonstrate low aspartate and high methylnicotinamide levels as well. We hypothesized that if these metabolites and pathways were biologically relevant, then similar changes might be identified in subjects who survived but demonstrated significant or prolonged illness in the postoperative period. We, therefore, performed an additional set of analyses, evaluating the ability to identify subjects with prolonged ICU LOS based on their early metabolic fingerprint and in particular based on their 24‐hour aspartate and methylnicotinamide levels.

**Figure 14:**
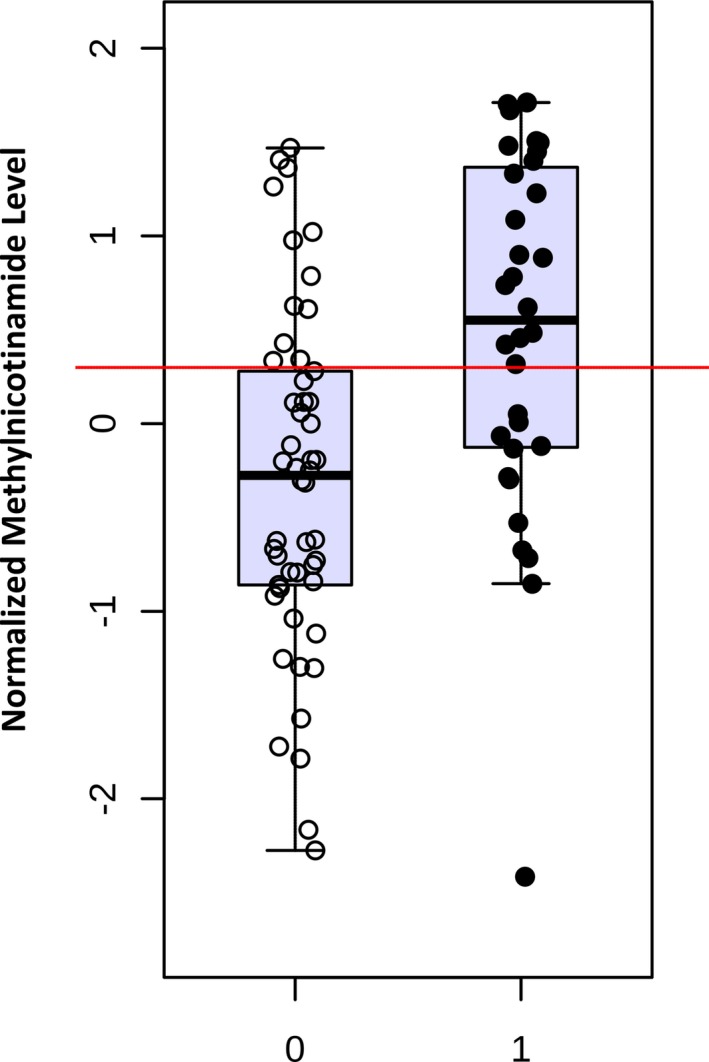
Normalized preoperative methylnicotinamide levels in subjects with lower (0) vs upper (1) 50% intensive care unit length of stay.

Metabolic fingerprinting demonstrated reasonable discrimination between subjects with upper versus lower 50% of ICU LOS (R^2^Y=0.94), with modest performance on cross‐validation (Q^2^Y=0.39). Affected metabolic pathways again included nicotinate/nicotinamide metabolism (*P*=3.4×10^−6^) and pathways involving aspartate metabolism (alanine/aspartate/glutamate metabolism [*P*=2.1×10^−4^], cysteine/methionine metabolism [*P*=2.7×10^−5^], pantothenate/coenzyme A biosynthesis [*P*=5.3×10^−5^], nitrogen metabolism [*P*=1.5×10^−4^], glycine/serine/threonine metabolism [*P*=2.8×10^−5^], and histidine metabolism [*P*=1.2×10^−4^]). Similar patterns of change in individual biomarkers were also noted, particularly decreased aspartate (*P*=5.8×10^−6^) (Figure 12B) and glutamate (*P*=2.2×10^−4^) and elevated methylnicotinamide (*P*=7.4×10^−7^) (Figure 13B), trigonelline (*P*=3.7×10^−5^), and kynurenic acid (*P*=1.6×10^−4^) in subjects with longer ICU LOS. On multivariable analysis controlling for relevant clinical variables, all 5 of these metabolites remained independently associated with ICU LOS. Again, the combination of 24‐hour aspartate and methylnicotinamide demonstrated the highest AUC to differentiate between subjects with lower and upper 50% for ICU LOS on permutation testing (AUC, 0.89; empiric *P*<0.01). This combination of metabolites demonstrated almost identical discriminative capacity when analyzed in neonates alone (AUC, 0.91; empiric *P*=0.01). Addition of an ordinal variable coding for neonate versus older infant directly to the multivariable biomarker analysis did not alter the discriminative capacity of these metabolites. Interestingly, although changes in aspartate appear to be a predominantly postoperative finding, methylnicotinamide levels also differed to a significant degree at the preoperative time point between subjects with lower and upper 50% for ICU LOS (*P*=0.0002) (Figure 14).

## Discussion

### Key Findings

Our results demonstrate the potential of novel metabolomics techniques to improve the understanding of physiological features and outcomes in infants undergoing cardiac surgery with CPB. Key findings of the study include a significant difference in the preoperative metabolic fingerprints of neonates compared with older infants and a marked postoperative metabolic shift that is similar in both neonates and older infants. In both the full cohort and the neonatal/older infant subcohorts, the metabolome continued to diverge from baseline through 24 hours after operation. Metabolic fingerprinting and specific metabolites at 24 hours showed significant potential to identify patients at risk for mortality and prolonged ICU LOS, although these findings require further validation. In particular, derangements in aspartate and methylnicotinamide metabolism discriminated between survivors and nonsurvivors as well as identified patients with prolonged ICU LOS.

### Preoperative Age Differences

We first sought to determine if our neonates demonstrated a different metabolic fingerprint compared with our older infants. This determination would then inform the potential need to analyze the neonates after stratification by age throughout the study. The first months of life are marked by significant physiological change, beginning with the transition away from placental supply of nutrition and oxygen and continuing through a period of rapid physical growth and maturation of endocrine, renal, and immune systems.^31^ It would be expected that these physiological changes might result in concurrent age‐related metabolomics changes, although the evolution of the metabolic fingerprint in early infancy remains only partially defined. Two studies have demonstrated a shift in urine^32^ and plasma^33^ metabolites in healthy infants beyond the neonatal period. Recently, Scalabre et al performed nuclear magnetic resonance spectroscopic analysis of urine from 90 healthy infants aged <4 months, including some neonates.^31^ They found significant associations between both age and growth and the infants’ urinary metabolic profiles, consistent with studies in older infants. The authors concluded that metabolomics studies in this population should account for differences in age and growth during analysis.

Our current findings demonstrate a substantial difference between the preoperative serum metabolic fingerprints of neonates and older infants with congenital heart disease. These differences were broad, affecting multiple pathways with significant differences in >25% of the measured metabolites. The most distinctive differences between the neonates and older infants in our study at the preoperative time point, including lower phosphorylcholine and higher indole‐3‐carboxylic acid, cystathionine, and cytidine in neonates, have not been previously identified and, to our knowledge, represent novel findings. Many of the other differential metabolites and metabolic pathways (tyrosine metabolism, purine metabolism, nitrogen metabolism, and arginine/proline metabolism) are similar to age‐related changes previously described in cohorts of older infants.^31^, ^32^, ^33^ A few metabolites, however (eg, succinate and methylnicotinamide), demonstrate strong differences between groups but with opposite directionality compared with prior studies of age‐related metabolic changes in older healthy infants.^32^ It is possible that these differences may be related to the increased proportion of young neonates (aged 1–7 days) in our study compared with prior studies or differences between serum and urine metabolites.^31^ Alternatively, disease‐specific factors, such as duration/severity of heart failure and cyanosis, may also contribute. Although further study is needed to better define age‐related changes in the circulating metabolome of young infants with congenital heart disease, on the basis of these findings, we concluded that age should be accounted for in our postoperative analysis.

### Postoperative Metabolomic Changes

Cardiac surgery with CPB results in both systemic and organ‐specific physiological changes, and it has been hypothesized that CPB also leads to comparable alterations in metabolic profiles.^34^ Changes in individual metabolites after pediatric cardiac surgery are well documented,^11^, ^12^, ^13^, ^14^, ^15^ but there are limited data addressing global changes in the postoperative metabolome. In the only study to explore this question to date, Correia et al used proton nuclear magnetic resonance spectroscopy of plasma specimens to evaluate circulating metabolic profiles of 28 children through 48 hours after operation.^3^ Subjects in this study had a median age of 6.6 months and a median risk‐adjusted congenital heart surgery score of 2 (low to moderate risk). The authors identified a significant shift in full, untargeted nuclear magnetic resonance profile (specific metabolites not identified) that, on average, peaked on admission to the ICU and returned toward baseline by 24 hours. One higher‐acuity patient demonstrated a progressive divergence from his or her baseline metabolic profile in the setting of severe postoperative illness.^3^

Our cohort demonstrated early postoperative changes similar to those found by Correia et al,^3^ with a strong shift in the metabolic fingerprint between baseline and rewarming from CPB. However, unlike the prior study cohort, our patients demonstrated a consistent, progressive divergence of their metabolic fingerprint from baseline through the 24‐hour time point. In our cohort, >84% of measured metabolites changed significantly between the preoperative time point and 24 hours postoperatively, a remarkably strong biological signal. Notably, unlike the preoperative metabolome, which varied distinctly by age, these postoperative changes were nearly identical in neonates and older infants. This progressive metabolic evolution was similar to the higher‐acuity patient described in the study by Correia et al^3^ and is consistent with the typical postoperative course after neonatal surgery because cardiac output nadirs and inflammatory response peak between 12 and 24 hours after surgery.^35^

Using our targeted screening platform, we were able to provide insight into some of the affected pathways and specific metabolites driving postoperative metabolomics changes in our cohort. Although multiple pathways were significantly altered in the postoperative period, we found the most marked changes in glutathione and amino acid metabolic pathways. Glutathione is a critical component of antioxidant defense and was elevated in the postoperative samples, consistent with prior studies in adult CPB.^36^ Amino acid metabolism appeared to be profoundly affected, with significant progressive decreases in multiple amino acids and associated intermediates from baseline to rewarming to 24 hours after operation. A decrease in glucogenic amino acids has previously been reported after adult cardiac surgery.^37^ Our cohort demonstrated decreases in almost all amino acids, similar to findings of a recent study on the effects of steroid administration in children undergoing cardiac surgery.^38^ This global decrease in amino acid levels is likely multifactorial. Generally, amino acid homeostasis is tightly preserved through reduced protein synthesis and autophagy, and short‐term fasting in older patients does not result in amino acid depletion.^39^ However, neonates and young infants frequently do not receive nutrition other than dextrose infusion for several hours before surgery as well as during the first 12 to 24 hours after operation and may not have the protein reserve to maintain homeostasis for that extended period of time. We also identified marked progressive increases in putrescine (a breakdown product of amino acids, particularly arginine)^40^ in 24‐hour postoperative samples, suggesting a potential added component of increased amino acid catabolism contributing to amino acid depletion. These findings suggest the possible need for early amino acid supplementation after complex infant cardiac surgery and warrant further study.

### Postoperative Outcomes

Assessing changes in postoperative metabolism may help identify opportunities to globally improve the care of this high‐risk group of children. Of potentially greater value, however, is the use of early pathologic metabolic fingerprints or specific metabolite biomarkers to identify groups of patients who will ultimately progress to poor outcomes. Our assessment of 24‐hour metabolic fingerprints by supervised dimension reduction techniques (PLS‐DA) demonstrated a modest ability to identify nonsurvivors as well as patients with prolonged ICU LOS within our specific cohort. However, these global assessments should be interpreted with caution. The metabolic response to cardiac surgery with CPB is so powerful and distinctive that it creates many similarities in the postoperative metabolic fingerprints of patients. In other words, infant cardiac surgery tends to make patients metabolically homogeneous in the immediate postoperative period. Therefore, attempting to evaluate subtle differences using global fingerprint analysis techniques is difficult and may be prone to overfitting, especially when evaluating rare outcomes, such as mortality.

Pathway analysis and specific biomarker testing, however, appear to be promising in this setting. Nonsurvivors demonstrated differences in nicotinamide metabolism as well as aspartate metabolism at 24 hours after operation. On additional analysis, we found that these specific pathways were also affected in subjects experiencing longer ICU LOS, suggesting potential biologic relevance of these pathways in the postoperative period. We are unable to determine with this study whether pathway disruption or deficiency/toxicity of individual metabolites is more relevant from a pathophysiological standpoint, and it is possible that both pathways and individual metabolites are important. It is interesting that among the top metabolites determining variation between survivors and nonsurvivors as well as ICU LOS, in 2 instances, pairs of metabolites (aspartate/glutamate and methylnicotinamide/trigonelline) share closely related pathways. Complete pathway mapping would be an important next step in determining the underlying cause of pathway disruption and changes in individual metabolite levels.

On an individual metabolite level, the 5 metabolites that most consistently differed in our sickest patients (decreased aspartate/glutamate and increased methylnicotinamide, trigonelline, and kynurenic acid) may have biologic relevance in this setting. Aspartate and glutamate serve as precursors, intermediates, or end products in multiple metabolic pathways, including nitrogen metabolism, coenzyme A biosynthesis, and amino acid metabolism pathways. They also have significant independent biological roles as signaling molecules and carriers. Aspartate and glutamate serve as the rate‐controlling step in Nicotinamide adenine dinucleotide transport into the mitochondria and are particularly important for cardiomyocyte energy production.^41^ Both glutamate and aspartate can also serve as neurotransmitters,^42^ and aspartate may have effects on central neurohormonal regulation, including stimulating vasopressin release.^43^ The normal response after adult cardiac surgery is a maintenance or increase in aspartate and glutamate,^44^ and it is possible that failure to maintain levels of these critical amino acids could contribute to energy failure and poor outcomes in the neonatal and infant population.

Until recently, methylnicotinamide and trigonelline (methylnicotinate) were thought to be biologically inactive intermediate metabolites of nicotinamide metabolism.^45^ Current studies have demonstrated significant positive effects by methylnicotinamide on endothelial production of prostacyclin and NO,^45^, ^46^ as well as potential anti‐inflammatory properties through the inhibition of reactive oxygen species.^47^ It is possible that increased methylnicotinamide represents an adaptive response to poor cardiac output and oxygen delivery. Alternatively, excessive production or reduced clearance of methylnicotinamide could, in theory, contribute to pathologic vasoplegia. Although less well studied, trigonelline also has the capacity to induce vasodilation through endothelial prostaglandin release.^48^ Interestingly, both metabolites have recently been shown to decrease in urine after ischemia‐reperfusion injury to murine kidneys, suggesting a potential cause of reduced renal clearance of these metabolites.^49^

Kynurenic acid is an intermediate of tryptophan catabolism and is associated with increased inflammatory activation and oxidative stress.^50^ Specifically, kynurenic acid is produced by irreversible transamination of tryptophan or kynurenine with reactive oxygen species and has multiple downstream functions, including as a reactive oxygen species scavenger, a noncompetitive antagonist of neuronal glutamate and acetylcholine receptors, and an immunosuppressive agent through G‐protein–coupled receptor 35.^51^ Kynurenic acid has been shown to increase after adult cardiac surgery,^52^ and elevated plasma kynurenic acid levels were found to be associated with severity of shock, early death, and poor long‐term outcome in adults after cardiac arrest.^53^ Preclinical data also suggest a role of elevated kynurenic acid in the impairment of cardiomyocyte mitochondrial function.^54^ As such, kynurenic acid may be both a marker of oxidative stress as well as a pathologic metabolite in this population.

### Directions for Future Research

In this study, we have demonstrated some of the vast potential for metabolomics analysis in cardiac surgery and postoperative critical care. Using targeted metabolic fingerprinting, we have identified potential age‐related differences in preoperative metabolism, a consistent shift in postoperative metabolism highlighted in our patients by a broad circulating amino acid deficiency, and as many as 5 candidate biomarkers for poor postoperative outcomes. In the long‐term, we are interested in identifying what, from a metabolic standpoint, sets up an individual patient to fail his or her surgical repair, allowing personalization of preoperative risk stratification and intraoperative/postoperative support as well as targeted metabolic therapeutics. Current challenges to realizing this goal include relatively narrow available targeted metabolic panels; dependence on relative quantification, as opposed to absolute quantification, of metabolites; and time/cost required to run metabolic panels by tandem mass spectrometry. Because specific metabolites of interest are identified, though, development of point‐of‐care testing for risk stratification, diagnosis, and therapeutic monitoring, on the basis of individual or small panels of metabolites, is highly feasible. And with ongoing improvement and miniaturization of technology, it is easy to imagine a time when continuous monitoring of pertinent panels of metabolites, similar to continuous glucose monitoring, could be performed during CPB or in the ICU to allow for real‐time maintenance of metabolic homeostasis during critical illness. Beyond diagnostics, advanced understanding of the metabolic shifts in critical illness will ultimately lead to development of novel metabolic‐driven therapies, either through reduction of toxic metabolites or treatment with beneficial metabolites aimed at improving outcomes of these high‐risk patients.

## Limitations

We acknowledge several limitations of our study. As a single‐center study, our findings may not be fully generalizable to other cardiac surgery centers because regional differences in perioperative care (including cardiovascular pharmaceutical support, fluid management, and nutrition) may result in differences in metabolic profiles. External validation is required to begin to understand the effects of these practice variations. The use of residual samples from a parent cohort study limited our sample size, could have introduced sampling bias, and limited our tracking of the metabolic fingerprint of these subjects to the first 24 hours after operation. Future longitudinal study of the metabolic fingerprint beyond 24 hours after operation, to at minimum cover the time of peak clinical derangement and postoperative organ dysfunction (48–72 hours), would substantially increase our understanding of postoperative metabolic recovery and add valuable information on prognostic metabolic profiling. Sample collection techniques could also affect the composition of the circulating metabolome. Although we chose to study a more generalizable and translatable collection method (standard serum processing), some classes of metabolites (eg, adenine nucleotides) would require special processing to capture an accurate picture of the steady‐state circulating metabolome.

From an analysis standpoint, moving toward targeted analysis has multiple advantages. In particular, using validated, targeted techniques increases certainty of metabolite identification compared with untargeted techniques and allows for greater translatability as well as increased analytic capacity (including pathway analysis). However, targeted analysis does run the risk of missing additional physiologically important metabolites that are either known but not measured or fully unknown. Even measuring 165 metabolites, we do not have full coverage of individual metabolic pathways and can still only assess which pathways are affected, but not provide complete pathway mapping. Additional studies are necessary to map individual candidate pathways to fully explore mechanisms behind modulation of these pathways. Ultimately, continued increase in the size of fully quantifiable, targeted metabolite panels is needed to maximize the information provided by this field. We are also limited to measuring the circulating metabolic fingerprint, and tissue‐level analysis in animal models or use of noninvasive magnetic resonance imaging–based technology would provide useful complementary information on organ‐specific metabolism. Finally, our sample size was sufficient to study groups of patients with large differences in metabolic fingerprints, such as neonates versus older infants or preoperative versus postoperative. Subtler differences or the study of rare outcomes, such as postoperative mortality, would benefit greatly from additional testing in larger validation cohorts.

## Conclusions

Neonates with congenital heart disease demonstrated large differences in their preoperative metabolic fingerprints compared with older infants. Postoperatively, all infants undergoing CPB demonstrated a marked shift in their metabolic fingerprint, highlighted by oxidative stress pathways and a broad amino acid deficiency. Nonsurvivors and subjects requiring longer ICU LOS showed changes in nicotinate/nicotinamide metabolism as well as pathways affected by aspartate metabolism. Several specific metabolite biomarkers, including aspartate, glutamate, methylnicotinamide, trigonelline, and kynurenic acid, may help differentiate these critical patients from patients experiencing a more benign course. Further research is needed to validate these findings and assess the diagnostic and therapeutic potential of these metabolites in infants undergoing cardiac surgery.

## Sources of Funding

This study is supported by National Institutes of Health (NIH)/National Heart, Lung, and Blood Institute 1K23HL123634 (principal investigator [PI] Davidson), American Heart Association 13CRP17300016 (PI Davidson), and NIH/National Center for Advancing Translational Sciences Colorado Clinical and Translational Science Award Grant UL1 TR001082 (University of Colorado). Contents are the authors’ sole responsibility and do not necessarily represent official NIH views. The funding agencies did not have any role in the study design, collection/analysis/interpretation of the data, the writing of the report, or the decision to submit the article for publication.

## Disclosures

Wischmeyer is an associate editor of *Clinical Nutrition* (Elsevier). He has received grant funding from the Canadian Institutes of Health Research (significant), Baxter (modest), Fresenius (modest), Lyric Pharmaceuticals (modest), Isomark Inc (modest), and Medtronics (modest). He has served as a consultant (all modest) to Abbott, Cardinal, Fresenius, Baxter, Medtronics, Nutricia, Lyric Pharmaceuticals, and Takeda for research related to metabolism and nutrition in illness. He has received honoraria or travel expenses (all modest) for lectures on improving nutrition and metabolic care in illness from Abbott, Fresenius, Cardinal, and Medtronics. None of these entities were related to or influenced the design, conduct, analysis, or manuscript preparation for this study. The remaining authors have no disclosures to report.

## References

[1] Bujak R , Struck‐Lewicka W , Markuszewski MJ , Kaliszan R . Metabolomics for laboratory diagnostics. J Pharm Biomed Anal. 2015;113:108–120. 25577715 10.1016/j.jpba.2014.12.017

[2] McGarrah RW , Crown SB , Zhang GF , Shah SH , Newgard CB . Cardiovascular metabolomics. Circ Res. 2018;122:1238–1258. 29700070 10.1161/CIRCRESAHA.117.311002 PMC6029726

[3] Correia GD , Wooi Ng K , Wijeyesekera A , Gala‐Peralta S , Williams R , MacCarthy‐Morrogh S , Jimenez B , Inwald D , Macrae D , Frost G , Holmes E , Pathan N . Metabolic profiling of children undergoing surgery for congenital heart disease. Crit Care Med. 2015;43:1467–1476. 25844698 10.1097/CCM.0000000000000982 PMC4467581

[4] Slaughter AL , Nunns GR , D'Alessandro A , Banerjee A , Hansen KC , Moore EE , Silliman CC , Nemkov T , Moore HB , Fragoso M , Leasia K , Peltz ED . The metabolopathy of tissue injury, hemorrhagic shock, and resuscitation in a rat model. Shock. 2018;49:580–590. 28727610 10.1097/SHK.0000000000000948 PMC5775055

[5] Tomic V , Russwurm S , Moller E , Claus RA , Blaess M , Brunkhorst F , Bruegel M , Bode K , Bloos F , Wippermann J , Wahlers T , Deigner HP , Thiery J , Reinhart K , Bauer M . Transcriptomic and proteomic patterns of systemic inflammation in on‐pump and off‐pump coronary artery bypass grafting. Circulation. 2005;112:2912–2920. 16275880 10.1161/CIRCULATIONAHA.104.531152

[6] Beghetti M , Rimensberger PC , Kalangos A , Habre W , Gervaix A . Kinetics of procalcitonin, interleukin 6 and C‐reactive protein after cardiopulmonary‐bypass in children. Cardiol Young. 2003;13:161–167. 12887072 10.1017/s1047951103000301

[7] Pavione MA , Carmona F , de Castro M , Carlotti AP . Late remote ischemic preconditioning in children undergoing cardiopulmonary bypass: a randomized controlled trial. J Thorac Cardiovasc Surg. 2012;144:178–183. 22244567 10.1016/j.jtcvs.2011.12.029

[8] Blinder JJ , Goldstein SL , Lee VV , Baycroft A , Fraser CD , Nelson D , Jefferies JL . Congenital heart surgery in infants: effects of acute kidney injury on outcomes. J Thorac Cardiovasc Surg. 2012;143:368–374. 21798562 10.1016/j.jtcvs.2011.06.021

[9] Shi S , Zhao Z , Liu X , Shu Q , Tan L , Lin R , Shi Z , Fang X . Perioperative risk factors for prolonged mechanical ventilation following cardiac surgery in neonates and young infants. Chest. 2008;134:768–774. 18625673 10.1378/chest.07-2573

[10] Typpo KV , Larmonier CB , Deschenes J , Redford D , Kiela PR , Ghishan FK . Clinical characteristics associated with postoperative intestinal epithelial barrier dysfunction in children with congenital heart disease. Pediatr Crit Care Med. 2015;16:37–44. 25162512 10.1097/PCC.0000000000000256 PMC4286428

[11] Alves RL , Aragao e Silva AL , Kraychete NC , Campos GO , Martins Mde J , Modolo NS . Intraoperative lactate levels and postoperative complications of pediatric cardiac surgery. Paediatr Anaesth. 2012;22:812–817. 22409574 10.1111/j.1460-9592.2012.03823.x

[12] Molina Hazan V , Gonen Y , Vardi A , Keidan I , Mishali D , Rubinshtein M , Yakov Y , Paret G . Blood lactate levels differ significantly between surviving and nonsurviving patients within the same risk‐adjusted classification for congenital heart surgery (RACHS‐1) group after pediatric cardiac surgery. Pediatr Cardiol. 2010;31:952–960. 20495912 10.1007/s00246-010-9724-7

[13] Greenberg JH , Zappitelli M , Devarajan P , Thiessen‐Philbrook HR , Krawczeski C , Li S , Garg AX , Coca S , Parikh CR ; TRIBE‐AKI Consortium . Kidney outcomes 5 years after pediatric cardiac surgery: the TRIBE‐AKI study. JAMA Pediatr. 2016;170:1071–1078. 27618162 10.1001/jamapediatrics.2016.1532 PMC5476457

[14] Agus MS , Asaro LA , Steil GM , Alexander JL , Silverman M , Wypij D , Gaies MG ; SPECS Investigators . Tight glycemic control after pediatric cardiac surgery in high‐risk patient populations: a secondary analysis of the safe pediatric euglycemia after cardiac surgery trial. Circulation. 2014;129:2297–2304. 24671945 10.1161/CIRCULATIONAHA.113.008124 PMC4043858

[15] Verhoeven JJ , Hokken‐Koelega AC , den Brinker M , Hop WC , van Thiel RJ , Bogers AJ , Helbing WA , Joosten KF . Disturbance of glucose homeostasis after pediatric cardiac surgery. Pediatr Cardiol. 2011;32:131–138. 21082177 10.1007/s00246-010-9829-z PMC3033526

[16] Davidson JA . Data files accompanying: metabolomic fingerprinting of infants undergoing cardiopulmonary bypass: changes in metabolic pathways and association with mortality and cardiac intensive care unit length of stay. 2018 Available at: https://www.researchgate.net/profile/Jesse_Davidson/publications. Accessed November 28, 2018. 10.1161/JAHA.118.010711 PMC6405618 30561257

[17] Davidson JA , Urban TT , Baird C , Tong S , Woodruff A , Twite M , Jaggers J , Simoes EAF , Wischmeyer P . Alkaline phosphatase in infant cardiopulmonary bypass: kinetics and relationship to organ injury and major cardiovascular events. J Pediatr. 2017;190:49e2–49e55.e2. 29144270 10.1016/j.jpeds.2017.07.035 PMC5726771

[18] Davidson JA , Urban T , Tong S , Twite M , Woodruff A , Wischmeyer PE , Klawitter J . Alkaline phosphatase, soluble extracellular adenine nucleotides, and adenosine production after infant cardiopulmonary bypass. PLoS One. 2016;11:e0158981. 27384524 10.1371/journal.pone.0158981 PMC4934870

[19] Davidson JA , Urban TT , Tong S , Maddux A , Hill G , Frank BS , Watson JD , Jaggers J , Simoes EAF , Wischmeyer P . Alkaline phosphatase activity and endotoxemia after infant cardiothoracic surgery. Shock 2018 Available at: https://insights.ovid.com/crossref?an=00024382-900000000-97841. Accessed November 28, 2018. 10.1097/SHK.0000000000001162 PMC6191388 29664834

[20] Yuan M , Breitkopf SB , Yang X , Asara JM . A positive/negative ion‐switching, targeted mass spectrometry‐based metabolomics platform for bodily fluids, cells, and fresh and fixed tissue. Nat Protoc. 2012;7:872–881. 22498707 10.1038/nprot.2012.024 PMC3685491

[21] Klepacki J , Klawitter J , Klawitter J , Karimpour‐Fard A , Thurman J , Ingle G , Patel D, Christians U. Amino acids in a targeted versus a non‐targeted metabolomics LC‐MS/MS assay: are the results consistent? Clin Biochem. 2016;49:955–961. 27288551 10.1016/j.clinbiochem.2016.06.002 PMC6047903

[22] Chong J , Soufan O , Li C , Caraus I , Li S , Bourque G , Wishart DS , Xia J . MetaboAnalyst 4.0: towards more transparent and integrative metabolomics analysis. Nucleic Acids Res. 2018;46:W486–W494. 29762782 10.1093/nar/gky310 PMC6030889

[23] Worley B , Powers R . Multivariate analysis in metabolomics. Curr Metabolomics. 2013;1:92–107. 26078916 10.2174/2213235X11301010092 PMC4465187

[24] Sabatine MS , Liu E , Morrow DA , Heller E , McCarroll R , Wiegand R , Berriz GF , Roth FP , Gerszten RE . Metabolomic identification of novel biomarkers of myocardial ischemia. Circulation. 2005;112:3868–3875. 16344383 10.1161/CIRCULATIONAHA.105.569137

[25] de Chao la Barca JM , Bakhta O , Kalakech H , Simard G , Tamareille S , Catros V , Callebert J , Gadras C , Tessier L , Reynier P , Prunier F , Mirebeau‐Prunier D . Metabolic signature of remote ischemic preconditioning involving a cocktail of amino acids and biogenic amines. J Am Heart Assoc. 2016;5:e003891 DOI: 10.1161/JAHA.116.003891. 27664804 PMC5079040

[26] Sansbury BE , DeMartino AM , Xie Z , Brooks AC , Brainard RE , Watson LJ , DeFilippis AP , Cummins TD , Harbeson MA , Brittian KR , Prabhu SD , Bhatnagar A , Jones SP , Hill BG . Metabolomic analysis of pressure‐overloaded and infarcted mouse hearts. Circ Heart Fail. 2014;7:634–642. 24762972 10.1161/CIRCHEARTFAILURE.114.001151 PMC4102656

[27] Bhargava P , Fitzgerald KC , Calabresi PA , Mowry EM . Metabolic alterations in multiple sclerosis and the impact of vitamin D supplementation. JCI Insight. 2017;2:95302. 28978801 10.1172/jci.insight.95302 PMC5841876

[28] Lacour‐Gayet F , Clarke D , Jacobs J , Gaynor W , Hamilton L , Jacobs M , Maruszewski B , Pozzi M , Spray T , Tchervenkov C , Mavroudis C . The Aristotle score for congenital heart surgery. Semin Thorac Cardiovasc Surg Pediatr Card Surg Annu. 2004;7:185–191. 15283368 10.1053/j.pcsu.2004.02.011

[29] Gaies MG , Gurney JG , Yen AH , Napoli ML , Gajarski RJ , Ohye RG , Charpie JR , Hirsch JC . Vasoactive‐inotropic score as a predictor of morbidity and mortality in infants after cardiopulmonary bypass. Pediatr Crit Care Med. 2010;11:234–238. 19794327 10.1097/PCC.0b013e3181b806fc

[30] Davidson J , Tong S , Hancock H , Hauck A , da Cruz E , Kaufman J . Prospective validation of the vasoactive‐inotropic score and correlation to short‐term outcomes in neonates and infants after cardiothoracic surgery. Intensive Care Med. 2012;38:1184–1190. 22527067 10.1007/s00134-012-2544-x PMC4984395

[31] Scalabre A , Jobard E , Demede D , Gaillard S , Pontoizeau C , Mouriquand P , Elena‐Herrmann B , Mure PY . Evolution of newborns’ urinary metabolomic profiles according to age and growth. J Proteome Res. 2017;16:3732–3740. 28791867 10.1021/acs.jproteome.7b00421

[32] Chiu CY , Yeh KW , Lin G , Chiang MH , Yang SC , Chao WJ , Yao TC , Tsai MH , Hua MC , Liao SL , Lai SH , Cheng ML , Huang JL . Metabolomics reveals dynamic metabolic changes associated with age in early childhood. PLoS One. 2016;11:e0149823. 26914934 10.1371/journal.pone.0149823 PMC4767415

[33] Jorgenrud B , Stene LC , Tapia G , Boas H , Pepaj M , Berg JP , Thorsby PM , Oresic M , Hyotylainen T , Ronningen KS . Longitudinal plasma metabolic profiles, infant feeding, and islet autoimmunity in the MIDIA study. Pediatr Diabetes. 2017;18:111–119. 26791677 10.1111/pedi.12360

[34] Kirov H , Schwarzer M , Neugebauer S , Faerber G , Diab M , Doenst T . Metabolomic profiling in patients undergoing off‐pump or on‐pump coronary artery bypass surgery. BMC Cardiovasc Disord. 2017;17:93. 28381258 10.1186/s12872-017-0518-1 PMC5381030

[35] Wernovsky G , Wypij D , Jonas RA , Mayer JE Jr , Hanley FL , Hickey PR , Walsh AZ , Chang AC , Castaneda AR , Newburger JW , Wessel DL . Postoperative course and hemodynamic profile after the arterial switch operation in neonates and infants: a comparison of low‐flow cardiopulmonary bypass and circulatory arrest. Circulation. 1995;92:2226–2235. 7554206 10.1161/01.cir.92.8.2226

[36] Turker FS , Dogan A , Ozan G , Kibar K , Erisir M . Change in free radical and antioxidant enzyme levels in the patients undergoing open heart surgery with cardiopulmonary bypass. Oxid Med Cell Longev. 2016;2016:1783728. 28101295 10.1155/2016/1783728 PMC5214539

[37] Nie LM , Xiao XJ , Liang MZ , Liu GJ . Impact of cardiopulmonary bypass on free amino acid spectrum in plasma for patients with prosthetic valve replacement during the perioperative period. Sichuan Da Xue Bao Yi Xue Ban. 2004;35:693–695. 15460422

[38] van Zwol A , Oosterloo NBC , de Betue CT , Bogers A , de Liefde II , Deutz NEP , Joosten KFM . Effects of glucocorticoids on serum amino acid levels during cardiac surgery in children. Clin Nutr ESPEN. 2018;23:212–216. 29460801 10.1016/j.clnesp.2017.09.012

[39] Broer S , Broer A . Amino acid homeostasis and signalling in mammalian cells and organisms. Biochem J. 2017;474:1935–1963. 28546457 10.1042/BCJ20160822 PMC5444488

[40] Schneider BL , Hernandez VJ , Reitzer L . Putrescine catabolism is a metabolic response to several stresses in Escherichia coli. Mol Microbiol. 2013;88:537–550. 23531166 10.1111/mmi.12207 PMC3633691

[41] Lu M , Zhou L , Stanley WC , Cabrera ME , Saidel GM , Yu X . Role of the malate‐aspartate shuttle on the metabolic response to myocardial ischemia. J Theor Biol. 2008;254:466–475. 18603266 10.1016/j.jtbi.2008.05.033 PMC2572303

[42] Ota N , Shi T , Sweedler JV . D‐aspartate acts as a signaling molecule in nervous and neuroendocrine systems. Amino Acids. 2012;43:1873–1886. 22872108 10.1007/s00726-012-1364-1 PMC3555687

[43] D'Aniello A . D‐aspartic acid: an endogenous amino acid with an important neuroendocrine role. Brain Res Rev. 2007;53:215–234. 17118457 10.1016/j.brainresrev.2006.08.005

[44] Jakob SM , Stanga Z . Perioperative metabolic changes in patients undergoing cardiac surgery. Nutrition. 2010;26:349–353. 20053534 10.1016/j.nut.2009.07.014

[45] Domagala TB , Szeffler A , Dobrucki LW , Dropinski J , Polanski S , Leszczynska‐Wiloch M , Kotula‐Horowitz K , Wojciechowski J , Wojnowski L , Szczeklik A , Kalinowski L . Nitric oxide production and endothelium‐dependent vasorelaxation ameliorated by N1‐methylnicotinamide in human blood vessels. Hypertension. 2012;59:825–832. 22353616 10.1161/HYPERTENSIONAHA.111.183210

[46] Nejabati HR , Mihanfar A , Pezeshkian M , Fattahi A , Latifi Z , Safaie N , Valiloo M , Jodati AR , Nouri M . N1‐methylnicotinamide (MNAM) as a guardian of cardiovascular system. J Cell Physiol. 2018;233:6386–6394. 29741779 10.1002/jcp.26636

[47] Biedron R , Ciszek M , Tokarczyk M , Bobek M , Kurnyta M , Slominska EM , Smolenski RT , Marcinkiewicz J . 1‐Methylnicotinamide and nicotinamide: two related anti‐inflammatory agents that differentially affect the functions of activated macrophages. Arch Immunol Ther Exp (Warsz). 2008;56:127–134. 18373238 10.1007/s00005-008-0009-2 PMC2766500

[48] Ross BM . Methylnicotinate stimulated prostaglandin synthesis in patients with schizophrenia: a preliminary investigation. Prostaglandins Leukot Essent Fatty Acids. 2018;136:99–102. 28552466 10.1016/j.plefa.2017.05.002

[49] Chihanga T , Ma Q , Nicholson JD , Ruby HN , Edelmann RE , Devarajan P , Kennedy MA . NMR spectroscopy and electron microscopy identification of metabolic and ultrastructural changes to the kidney following ischemia‐reperfusion injury. Am J Physiol Renal Physiol. 2018;314:F154–F166. 28978534 10.1152/ajprenal.00363.2017 PMC5866452

[50] Wang Q , Liu D , Song P , Zou MH . Tryptophan‐kynurenine pathway is dysregulated in inflammation, and immune activation. Front Biosci (Landmark Ed). 2015;20:1116–1143. 25961549 10.2741/4363 PMC4911177

[51] Wirthgen E , Hoeflich A , Rebl A , Gunther J . Kynurenic acid: the Janus‐faced role of an immunomodulatory tryptophan metabolite and its link to pathological conditions. Front Immunol. 2017;8:1957. 29379504 10.3389/fimmu.2017.01957 PMC5770815

[52] Kotlinska‐Hasiec E , Nowicka‐Stazka P , Parada‐Turska J , Stazka K , Stazka J , Zadora P , Dabrowski W . Plasma kynurenic acid concentration in patients undergoing cardiac surgery: effect of anaesthesia. Arch Immunol Ther Exp (Warsz). 2015;63:129–137. 25205210 10.1007/s00005-014-0312-z PMC4359282

[53] Ristagno G , Latini R , Vaahersalo J , Masson S , Kurola J , Varpula T , Lucchetti J , Fracasso C , Guiso G , Montanelli A , Barlera S , Gobbi M , Tiainen M , Pettila V , Skrifvars MB ; FINNRESUSCI Investigators . Early activation of the kynurenine pathway predicts early death and long‐term outcome in patients resuscitated from out‐of‐hospital cardiac arrest. J Am Heart Assoc. 2014;3:e001094 DOI: 10.1161/JAHA.114.001094. 25092787 PMC4310405

[54] Baran H , Staniek K , Kepplinger B , Gille L , Stolze K , Nohl H . Kynurenic acid influences the respiratory parameters of rat heart mitochondria. Pharmacology. 2001;62:119–123. 11174083 10.1159/000056082

